# Differential diagnosis of Huntington’s disease− neurological aspects of *NKX2-1*-related disorders

**DOI:** 10.1007/s00702-024-02800-3

**Published:** 2024-06-25

**Authors:** Julia Skwara, Maciej Nowicki, Lucia Sharif, Łukasz Milanowski, Jarosław Dulski, Ewelina Elert-Dobkowska, Katarzyna Skrzypek, Dorota Hoffman-Zacharska, Dariusz Koziorowski, Jarosław Sławek

**Affiliations:** 1 Student’s Scientific Group, Department of Neurology, Faculty of Health Sciences, Medical University of Warsaw, Warsaw, Poland; 2 Department of Neurology, Faculty of Health Sciences, Medical University of Warsaw, Ludwika Kondratowicza 8, Warsaw, 03-242 Poland; 3 Department of Neurology and Stroke, St. Adalbert Hospital, Gdańsk, Poland; 4 Department of Neurology, Mayo Clinic, Jacksonville, FL USA; 5 Department of Genetics, Institute Psychiatry and Neurology, Warsaw, Poland; 6 Department of Medical Genetics, Institute of Mother and Child, Warsaw, Poland; 7 Division of Neurological and Psychiatric Nursing, Faculty of Health Sciences, Medical University of Gdansk, Gdańsk, Poland

**Keywords:** Benign hereditary chorea, Brain-lung-thyroid syndrome, Dystonia, Huntington’s disease, *NKX2-1*, *NKX2-1*-related disorders

## Abstract

Benign hereditary chorea (BHC) is an inherited neurological disorder consisting of childhood-onset, nonprogressive chorea, generally without any other manifestations. In most reported cases, the inheritance of BHC is autosomal dominant but both incomplete penetrance and variable expressivity are observed and can be caused by *NKX2-1* mutations. The spectrum contains choreoathetosis, congenital hypothyroidism, and neonatal respiratory distress syndrome. The neurological symptoms can be misdiagnosed as Huntington’s disease (HD). The two Polish families were diagnosed with *NKX2-1* gene mutations and a literature review concerning the *NKX2-1*-related disorders was conducted. All family members were examined by experienced movement disorders specialists. PubMed database was searched to obtain previously described *NKX2-1* cases. Whole exome sequencing (WES) was performed in one proband (Family A) and direct *NKX2-1* sequencing in the second (Family B). Two Polish families were diagnosed with *NKX2-1* gene mutations (p.Trp208Leu and p.Cys117Alafs*8). In one family, the co-occurrence of HD was reported. Forty-nine publications were included in the literature review and symptoms of 195 patients with confirmed *NKX2-1* mutation were analyzed. The most common symptoms were chorea and choreiform movements, and delayed motor milestones. The *NKX2-1* mutation should always be considered as a potential diagnosis in families with chorea, even with a family history of HD. Lack of chorea does not exclude the *NKX2-1-*related disorders.

## Introduction

Chorea can be a symptom of a variety of diseases, including hereditary neurological disorders, and their list is constantly increasing due to the availability of advanced molecular diagnostic techniques. The discovery of new genes, followed by the investigation of further cases with partially described phenotypes, often leads to the recognition of additional aspects of the disorders. Chorea is the major phenotypic aspect of Huntington’s disease (HD) (OMIM #143,100) as well as other Huntington-like syndromes and benign hereditary chorea (BHC) – a clinical manifestation of the *NKX2-1*-related disorders.

BHC is a rare autosomal dominant disorder. It was linked to chromosome 14q13 and a mutation in the *NKX2-1* gene (OMIM *600,635) was identified as a causative factor ending the controversy about the existence of BHC as a separate entity (Fernandez et al. 2001; Kleiner-Fisman et al. 2003). BHC usually has a childhood-onset, nonprogressive, with involuntary jerk-like movements, and sometimes with a tendency to improve in adulthood.

**Fig. 1:**
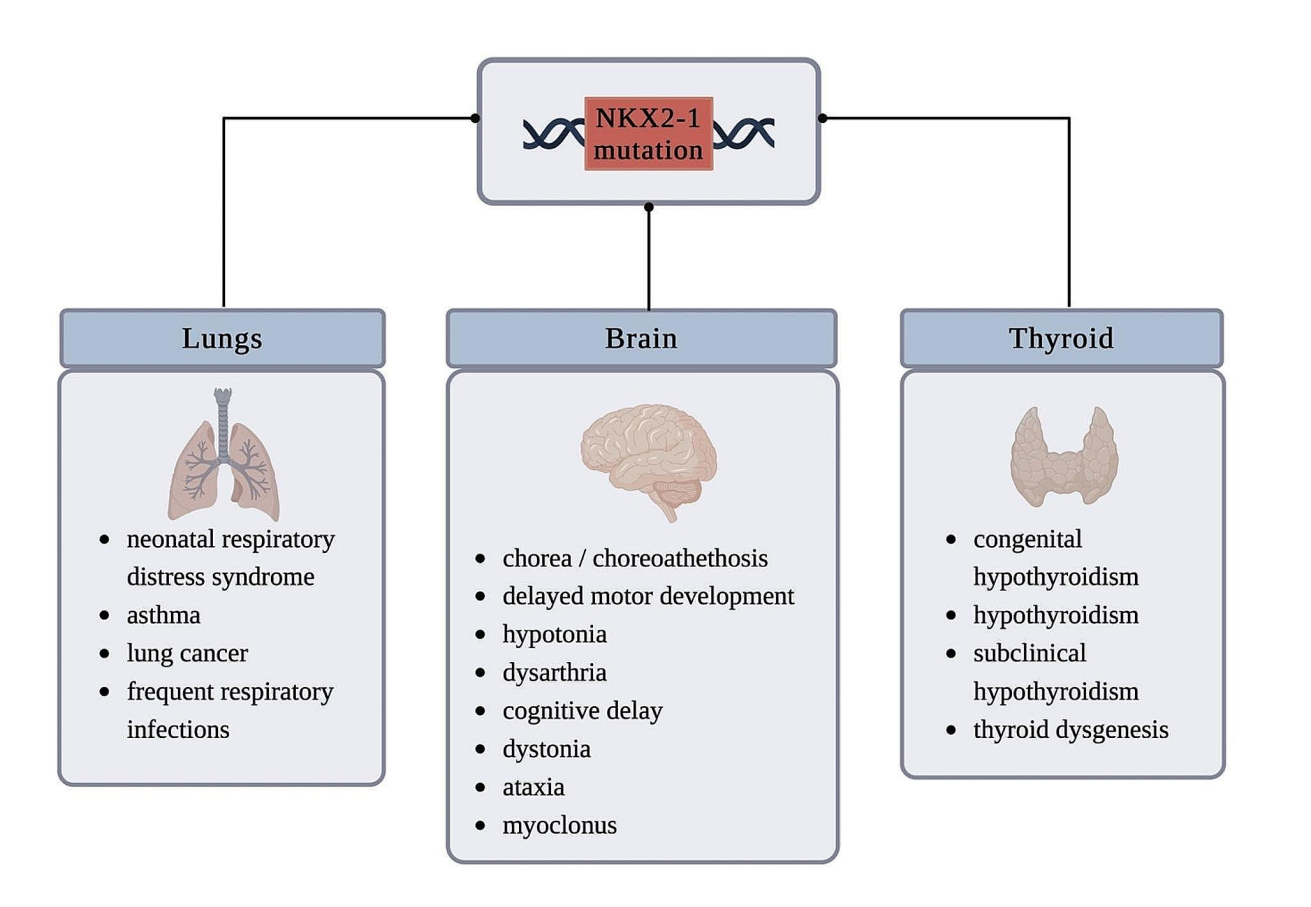
Phenotypes of *NKX2-1*-related disorders. It may manifest in one organ, or any combination, with all three being the “brain-lung-thyroid” syndrome. Created with BioRender.com

However, it is not always benign and the spectrum of manifestations is wide and complex. Neurological symptoms may manifest as chorea, choreoathetosis, developmental delay, cognitive deficits, hypotonia, myoclonus, ataxia, drop attacks, dystonia, and psychiatric disorders. Pulmonary dysfunction, which include respiratory distress syndrome and interstitial lung disease are the second most common manifestation (Gras et al. 2012). Patients might also present with congenital or compensated hypothyroidism due to thyroid dysfunction (Fig. 1).

The *NKX2-1*-related disorders are diagnosed by identifying a heterozygous pathogenic variant in the *NKX2-1* gene (Patel and Jankovic 2014 [updated 2023]). Both sporadic and familial forms have been reported. According to OMIM, both BHC (OMIM **#**118,700), chorea, congenital hypothyroidism with or without pulmonary dysfunction (CAHTP, OMIM #610,978); and susceptibility to nonmedullary thyroid cancer-1 (NMTC1, OMIM #188,550), are related to mutations in the *NKX2-1* gene.

In the Human Gene Mutation Database (HGMD Professional v.2024.1, 04.2024) 187 pathogenic/likely pathogenic mutations have been described. Mutations are dominant and haploinsufficiency due to loss of function is accepted as a mechanism of disease causation.

The *NKX2-1* gene encodes Homeobox protein Nkx-2.1 (alternatively named Thyroid transcription factor 1, TTF-1; UniProt, https://www.uniprot.org/uniprotkb/P43699) which is expressed during early development of thyroid, lung, and forebrain regions, particularly the basal ganglia and hypothalamus. The most frequently reported symptoms are related to the central nervous system, lungs, and thyroid gland, hence the clinical term *NKX2-1*-related disorders - “brain-lung-thyroid syndrome”. According to the available data, among individuals affected with *NKX2-1-*related disorders, 50% had the full brain-lung-thyroid syndrome, 30% brain and thyroid involvement, and 13% chorea only (Carré et al. 2009; Parnes et al. 2019).

The Nkx2-1 protein expression is modulated by various regulatory factors. What is more, this protein as nuclear transcription factor is involved in the regulation of expression of other genes necessary in the lung, thyroid and nervous system.

**Fig. 2:**
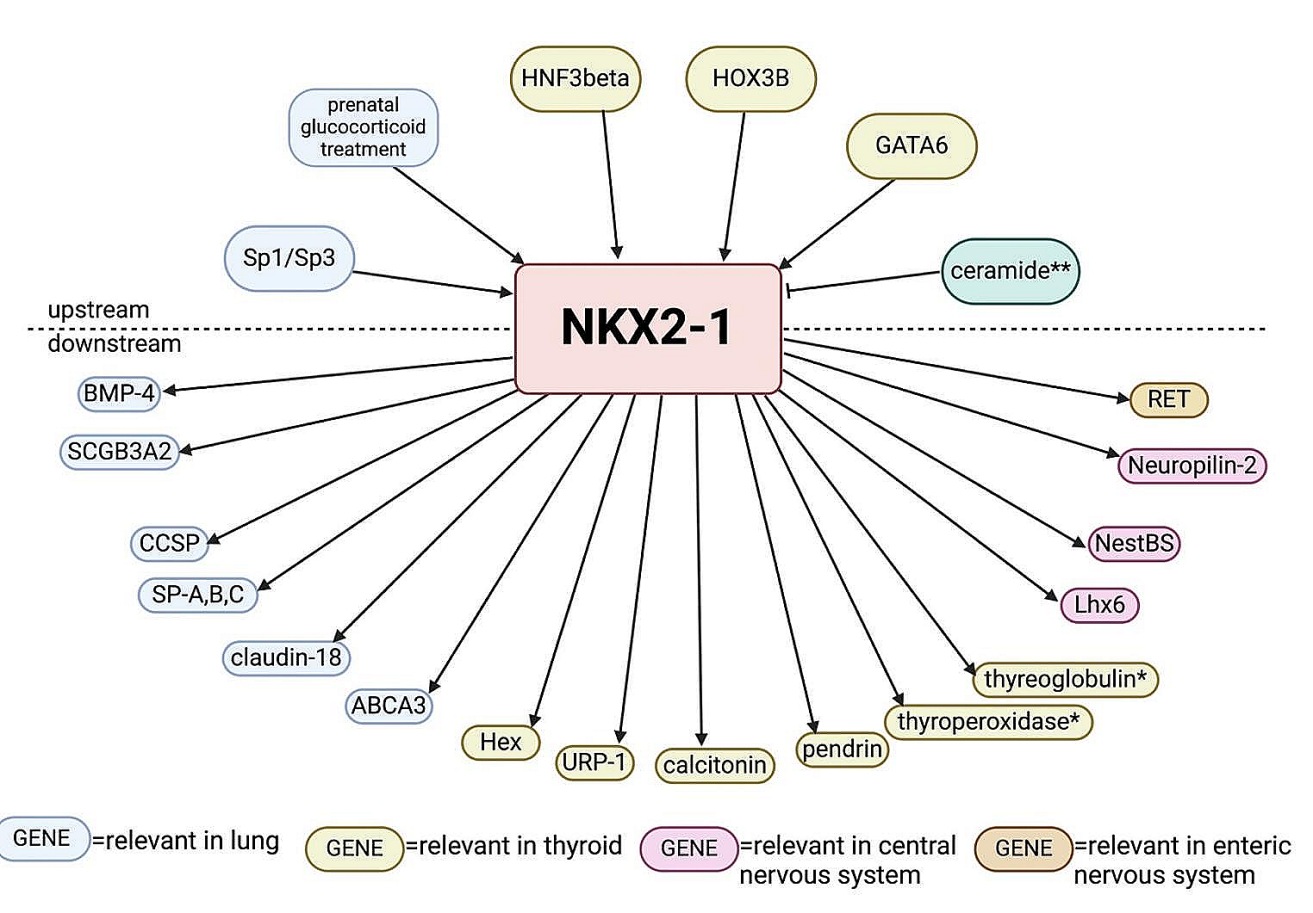
Upstream and downstream targets of NKX2-1. * activation in cooperation with PAX8. ** inhibits NKX2-1 in inflammation. SP, surfactant protein; CCSP, clara cell secretory protein; BMP-4, bone morphogenic protein 4; URP-1, uteroglobin related protein 1; NestBS, nestin binding site; ABCA, ATP-binding cassette sub-family A; RET, rearranged during transfection gene; SCGB3A2, secretoglobin 3A2. Based on Atlas of Genetics and Cytogenetics in Oncology and Haematology (Wilbertz et al. 2010). Created with BioRender.com

The TIF-1 protein cooperates with a lot of hormones and cytokines, such as thyroid stimulating hormone (TSH), nuclear factor (NFI) (Nakazato et al. 2000), hepatocyte nuclear factor-3 (HNF-3β) (Ikeda et al. 1996), SMAD family member 2 (Smad2) (Li et al. 2013), forkhead box protein A1 (FOXA1) (Minoo et al. 2007), forkhead box protein P2 (FOXP2) (Zhou et al. 2008), GATA-binding factor 6 (GATA6) (Yin et al. 2006) and TTF1 itself (Oguchi and Kimura 1998). TSH when binding to its thyroid stimulating hormone receptor (TSHR) activates the cAMP/PKA (cyclic adenosine monophosphate/protein kinase A) pathway, which enhances the *NKX2-1* expression as well as its activity (Guan et al. 2021) (Fig. 2).

This paper describes two Polish families with *NKX2-1* gene mutations (p.Trp208Leu and p.Cys117Alafs*8) and gathers data about other previously reported cases of patients with *NKX2-1* mutation that presented neurological disorders. The report pays particular attention to a rare case of familial co-occurrence of HD and *NKX2-1*-related disorders, which could be distinguished based on molecular tests.

## Materials and methods

### Clinical and genetic analysis

One family member from Family A and three from Family B of Polish origin were recruited from the Division of Neurological and Psychiatric Nursing, Faculty of Health Sciences, Medical University of Gdansk and Neurology Dpt., St. Adalbert Hospital in Gdansk (Family A) Department of Neurology, Faculty of Health Sciences, Medical University of Warsaw, in Warsaw (Family B), Poland.

The clinical diagnosis was established by two experienced movement disorders specialists (DK and JS) and blood samples were collected from the patients. Whole exome sequencing - WES (SureSelect Human All Exon v6 enrichment, Illumina NovaSeq 6000 platform, annotations according to Department of Medical Genetics, Institute of Mother and Child pipeline, VEP2.7) was performed for probands from Family B. The cosegregation of nominated variants was confirmed with Sanger sequencing in the two affected family B members (proband’s mother and brother). In proband from Family A direct Sanger sequencing of the *NKX2-1* gene (exons, exons/introus boundaries) was performed.

*HTT* (CAG)n repeat numbers were analysed using the standard procedure (Warner et al. 1993). PCR was performed with fluorescently labelled primers and 4% polyacrylamide gel electrophoresis on an ABI Prism 377 plate sequencer (Applied Biosystems).

### NKX2-1 variants reporting

There are two highly conserved *NKX2-1* transcripts. The longer mRNA-isoform 1 NM_001079668 contains exons 1, 2, and 3, whereas mRNA-isoform 2 NM_003317 contains only exons 2 and 3. Isoform 2 is the predominant and isoform 1 represents the minor transcript. However, because the most pathogenic variants in *NKX2-1* have been reported in this transcript the described variants are reported in relation to GRCh38 NM_001079668.3 MANE Select reference sequence (https://www.ncbi.nlm.nih.gov/nuccore/NM_001079668.3) and according to HGVS nomenclature recommendation v.21.0.2 (https://hgvs-nomenclature.org/stable/).

### Data analysis

PubMed database was searched using the following keywords; “benign hereditary chorea”, “TTF-1”, “brain-lung-thyroid syndrome”, “NKX2-1”, “chorea”, “choreoathetosis”, “ataxia”, “dystonia”, “neurology”, and “myoclonus”. A total of 49 articles were selected and included in this analysis.

## Results

### Presentation of cases

#### Family A

**Fig. 3:**
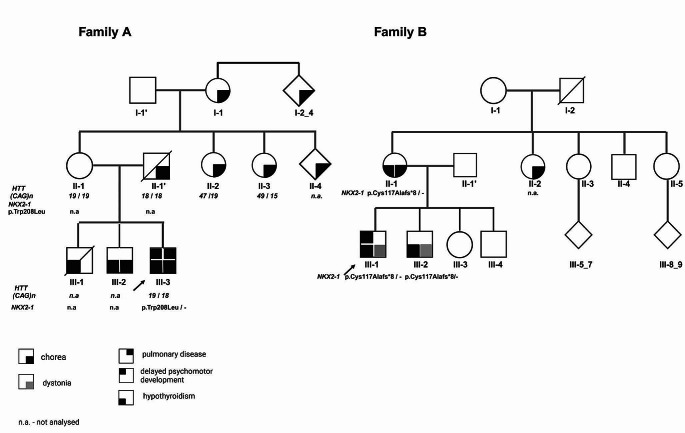
Pedigree of two described families carrying the NKX2-1 mutations Family A (p.Thr208Leu) and Family B (p.Cys117Alafs*8).

Proband (III-3) is a male patient who was diagnosed with infant hypotonia and delayed psychomotor development (Video 1). He could sit and walk at 2 and 5 years, respectively, and presented with mild cognitive impairment. At 5 years of age, he developed nonprogressive, generalized, mild chorea and dystonia followed by ataxia. He was also diagnosed with apathy and depression, hypothyroidism, and recurrent respiratory tract infections complicated by pneumothorax. He was initially treated with sulpiride, tiapride, and pridinol. However, these were discontinued due to their side effects. A trial with L-Dopa to exclude levodopa responsive dystonia was ineffective. He benefitted only from clonazepam. Brain MRI showed frontotemporal and cerebellar atrophy. He is still independent in his daily activities. The proband had two older brothers; the oldest (III-1) was diagnosed with a congenital heart defect and hypothyroidism and died when he was 6 months old, whereas the second brother (III-2) presented with chorea when he was 3 years old accompanied by hypothyroidism. There was a positive family history of similar symptoms on both the maternal and paternal side of the family. The proband’s father (II-1) showed generalized chorea with childhood-onset, developed alcoholism in adulthood, and died of colorectal cancer. The proband’s mother was healthy; however, her three siblings (II-2-4) presented with chorea and were diagnosed with HD, as well as proband’s grandmother (I-1) and her siblings (I-2-4). The molecular analysis of the *HTT* in subjects II-2 and II-3 revealed the presence of (CAG)n repeats in the pathogenic range (genotypes: 47/19 and 49/15) and these results confirmed clinical diagnosis. The analysis of *HTT* gene (CAG)n expansion was also performed for proband’s mother II-1 (carrier status) and father II-1’ (diagnostic test) and the results were negative (Fig. 3). Wilson disease was molecularly excluded for the proband. Subsequently, the analysis of *NKX2-1* gene revealed a presence of heterozygous substitution c.623G > T causing the missense variant p.(Trp208Leu), classified as pathogenic (Table 1). The identified variant, was already known and described in databases as pathogenic (HGMD 2024.1; accession: CM020775) causative for BHC (Breedveld et al. 2002) and pathogenic/likely pathogenic (ClinVar ID:8974 accession: VCV000008974.8) causative for BHC/Brain-lung-thyroid syndrome.

**Table 1:** *NKX2-1* variants characteristic

Fam	c.DNA NM_001-79668.3	Protein NP_001073136.1	ACMG criteria	CADD	Missense prediction		gnomAD frequency (v.3.1.2)	Final pathogenicity Classification*	Ref.
					MutTaster	PolyPhen2 (HumVar)			
A	c.623G > T	p.(Trp208Leu)	P	29.7	Deleterious	Probably Damaging	0	Pathogenic	Breedveld (2002)
B	c.348del	p.(Cys117Alafs*8)	LP	38.0	-	-	0	Pathogenic	-

P – pathogenic. LP – likely pathogenic

* interpretation of pathogenicity based on all analyzed criteria

During the 20-year follow-up, the proband’s symptoms did not progress and he remains independent in the activities of daily living at the age of 42.

#### Family B

A 29-year-old proband (III-1) presented with generalized dystonia affecting the face, neck, trunk, limbs (more affected on the left side than the right side), and slight dysarthria. There was no improvement after alcohol usage. He was born at term from an uneventful pregnancy. The involuntary movements began in early childhood and were first recognized as myoclonus. Developmental delay was observed in this patient in his. He was diagnosed with mild mental impairment. (He also suffered from hypothyroidism and depression. He was first admitted to the Department of Neurology when he was 18 years old. MRI was impossible to perform. His younger brother (III-2) had right limb dystonia and hypothyroidism. His mother (II-1) had cervical dystonia from early childhood with lower limb chorea and hypothyroidism. Analysis of the *TOR1A, THAP1*, and *SGCE* genes excluded dystonia type 1, 6 (DYT1, DYT6) and myoclonus dystonia (DYT11). WES revealed the presence of heterozygous deletion c.348delC, causing the frameshift variant p.(Cys117Alafs*8). This variant is new and has not been described previously. Because the mutation is a truncating variant in a gene where loss of function (LOF) is the mutational mechanism, and due to its cosegregation with the disease phenotype in the family (Fig. 3), this variant was recognized as likely pathogenic according to ACMG guidelines. Consequently, it was classified as pathogenic (Table 1). To exclude levodopa-responsive dystonia, the proband’s treatment was started with levodopa 300 mg with no improvement in the involuntary movements. His dystonia was treated with botulinum toxin injections and finally with globus pallidus DBS when he was 23 years old with a good response (Online Resource 1). This was the first DBS treatment of a *NKX2-1*-related dystonia.

### *NKX2-1-* related disorders – literature review

The literature search identified 49 publications that described 195 patients with confirmed *NKX2-1* gene mutations (Krude et al. 2002; Kawano et al. 2003; Doyle et al. 2004; Willemsen et al. 2005; Asmus et al. 2005, 2007; Costa et al. 2005; Moya et al. 2006; Glik et al. 2008; Provenzano et al. 2008, 2016; Nagasaki et al. 2008; Ferrara et al. 2008, 2012; Carré et al. 2009; Salvatore et al. 2010; Armstrong et al. 2011; Gras et al. 2012; Fons et al. 2012; Teissier et al. 2012; Konishi et al. 2013; Peall et al. 2014; McMichael et al. 2013; Nettore et al. 2013; Shetty et al. 2014; Thorwarth et al. 2014; Veneziano et al. 2014; Rosati et al. 2015; Williamson et al. 2014; de Gusmao et al. 2016; Monti et al. 2015; Tozawa et al. 2016; Koht et al. 2016; Shiohama et al. 2018; Gauquelin et al. 2017; Blumkin et al. 2018; Tübing et al. 2018; Parnes et al. 2019; Basu et al. 2018; Villafuerte et al. 2018; Iodice et al. 2019; Balicza et al. 2018; Gonçalves et al. 2019; Milone et al. 2019; Prasad et al. 2019; Graziola et al. 2021; Liao et al. 2021; Thust et al. 2022; Lamiral et al. 2020). The two probands were also added to this review study (Table 2).

**Table 2:** Clinical characteristics of our two probands and percentage analysis of all affected individuals found in the literature (including probands). *n* = 197

Clinical feature	Proband 1	Proband 2	% of affected individuals
Chorea and choreiform movements	**+**	**-**	84.3%
Delayed motor development (milestones)	**+**	**+**	81.2%
Hypotonia	**+**	**-**	47.7%
Dysarthria	**+**	**+**	25.4%
Cognitive delay and learning difficulties	**+**	**+**	24.9%
Dystonia	**+**	**+**	23.9%
Ataxia	**+**	**-**	23.4%
Myoclonus	**-**	**+**	14.7%
Choreoathetosis	**-**	**-**	10.2%
Abnormalities in MRI	**+**	**n/a**	20.2%*
Thyroid manifestation	**+**	**+**	67%
Lung manifestation	**+**	**-**	48.2%

* out of 104 patients who had an available MRI

Most of the analyzed patients (81.2%) had delayed motor milestones and a significant group (24.9%) had cognitive impairment. Only 3% of the described patients had an intellectual disability. Chorea and choreiform movements occurred in 84.3% of the patients, making it the most common symptom of *NKX2-1-*related disorders, which is why it is described in the literature as benign hereditary chorea. However, there are other neurological features that the patient can present, which makes the term “benign hereditary chorea” quite inaccurate since symptoms such as hypotonia (47.7%), dysarthria (25.4%), dystonia (23.9%), ataxia (23.4%), myoclonus (14.7%), and choreoathetosis (10.2%) can occur, sometimes in one patient.

In some cases, ataxia and ataxic gait were the symptoms preceding actual choreiform movements during infancy and childhood. There are examples of patients in which ataxia completely ended and was replaced by chorea (McMichael et al. 2013).

Moreover, there is an example in the literature of a 49-year-old patient with the *NKX2-1* mutation who presented with ataxic gait, but chorea had still not occurred. Therefore, *NKX2-1*-related disorders could be mistaken for ataxic cerebral palsy (McMichael et al. 2013).

8% of analyzed patients had attention-deficit hyperactivity disorder (ADHD), a notably high percentage that merits consideration of the potential interrelationship with the *NKX2-1* mutation. Several neuropsychiatric symptoms and disorders occurred in these analyzed cases, such as depression(Liao et al. 2021; Balicza et al. 2018; Salvatore et al. 2010; Ferrara et al. 2012), psychosis (Glik et al. 2008; Salvatore et al. 2010; Ferrara et al. 2012), schizophrenia (Glik et al. 2008), autism spectrum disorder (Milone et al. 2019; Shetty et al. 2014), anxiety (Balicza et al. 2018; Basu et al. 2018), obsessive-compulsive disorder (Peall et al. 2014; Parnes et al. 2019), conduct disorder (Liao et al. 2021) and disruptive behavior disorder (Liao et al. 2021).

Some individuals (3.6%) had a history of seizures (epileptic or non-epileptic). Loss of *NKX2-1* gene function in a mouse model during early neurogenesis causes seizures and hyperkinetic movements, resulting from a reduction of GABAergic cortical interneurons (Ferrara et al. 2012).

Other rare symptoms described in individual cases include apraxia (Veneziano et al. 2014; Thust et al. 2022; Salvatore et al. 2010), spasticity (Costa et al. 2005; Balicza et al. 2018; Liao et al. 2021), tics (Rosati et al. 2015; Gras et al. 2012; Koht et al. 2016), dysdiadochokinesis (Veneziano et al. 2014; Thust et al. 2022), dysmetria (Tübing et al. 2018; Provenzano et al. 2016; Gonçalves et al. 2019), stuttering (Costa et al. 2005; Ferrara et al. 2012; Koht et al. 2016), tremor (Liao et al. 2021; Williamson et al. 2014; Peall et al. 2014; Glik et al. 2008; Gras et al. 2012), nystagmus (Provenzano et al. 2016; Liao et al. 2021), dyslexia (Balicza et al. 2018) and restless leg syndrome (Iodice et al. 2019). Further studies are required to determine the linkage between these symptoms and *NKX2-1* pathogenic variants.

#### Thyroid manifestation

67% of patients had a thyroid manifestation of the disorder, such as congenital hypothyroidism, subclinical hypothyroidism, or thyroid dysgenesis.

#### Lung manifestation

More than 48% of patients had a lung manifestation of the disorder, such as neonatal respiratory distress syndrome, asthma, frequent respiratory infections, or lung cancer.

#### MRI

In most analyzed cases, magnetic resonance imaging (MRI) of the brain showed no abnormalities. However, 20.2% of patients who had an MRI had structural brain anomalies, such as cavum septum pellucidum (Balicza et al. 2018); hypoplastic pallidum (Krude et al. 2002); agenesis of the corpus callosum (Carré et al. 2009); ventricular dilatation (Salvatore et al. 2010); hippocampal dysmorphism (Iodice et al. 2019); Chiari malformation type 1 (Gonçalves et al. 2019); or mild cerebellar atrophy (Provenzano et al. 2016; Costa et al. 2005). Pituitary anomalies occurred in 61.9% of patients that had an abnormal MRI, such as abnormal sella turcica (Krude et al. 2002); empty sella (Balicza et al. 2018; Salvatore et al. 2010); pituitary cysts (Thust et al. 2022; Krude et al. 2002; Veneziano et al. 2014); anterior displacement of pituitary stalk and gland (Thust et al. 2022); or decreased size of the pituitary gland (Prasad et al. 2019; Iodice et al. 2019).

#### Other non-neurological manifestations

Single articles describe various symptoms that occur in patients with *NKX2-1* gene mutation: erectile dysfunction (Balicza et al. 2018); **i**mmunodeficiency (Villafuerte et al. 2018); ligamentous laxity (Villafuerte et al. 2018; Peall et al. 2014; Parnes et al. 2019); pes cavus (Costa et al. 2005; Peall et al. 2014); genitourinary abnormalities (Ferrara et al. 2008; Salvatore et al. 2010); congenital heart defect (Thorwarth et al. 2014); or hypodontia (Villafuerte et al. 2018). Further studies are required to determine the significance of these findings.

### Differential diagnosis

In some cases, primarily diagnosed with BHC, the HD, myoclonic dystonia, hereditary essential myoclonus or tics were found (Schrag et al. 2000). Chorea minima, physiologic chorea of infancy, idiopathic chorea or oro-bucco-lingual dyskinesia should be considered in the differential diagnosis in individuals with chorea characterized as childhood-onset and nonprogressive. Disorders such as ADCY5-associated disease; tumor-related chorea; Wilson disease; developmental chorea; Lesch-Nyhan syndrome; cerebral palsy or others where chorea is associated with progressive neurological and cognitive dysfunction can be differentiated from those related to *NKX2-1* mutations (Patel and Jankovic 2014 [updated 2023]).

#### Huntington’s disease

Choreiform movements in HD progress, in contrast to BHC, where they are described as nonprogressive. HD leads to marked cognitive dysfunction whereas in BHC, the patients’ cognition remains normal or only slightly impaired (Kleiner-Fisman 2011) (Table 3).

**Table 3:** Chorea – differential diagnosis. Based on a review article by Termsarasab (2019)

	Disorder	Onset of chorea*	Cause/pattern	Duration
Hereditary causes of chorea	Huntington disease	Adult	HTT gene mutation Autosomal dominant	chronic
	Huntington disease like-2 (HDL-2)	Adult	JPH3 gene mutation Autosomal dominant	
	Chorea-acanthocytosis	Adult	VPS13A Autosomal recessive	
	C9orf72 disease	Adult	C9orf72 gene mutation Autosomal dominant	
	Wilson disease	childhood	ATP7B gene mutation Autosomal recessive	
	Lesch-Nyhan syndrome	childhood	HPRT1 gene mutation X-linked recessive	
	*NKX2-1*-related disorders	childhood	NKX2-1 gene mutation Autosomal dominant	
	ADCY5-related dyskinesia	childhood	ADCY5 gene mutation Autosomal dominant	
Acquired causes of chorea	endocrine/metabolic	sporadic	e.g. hypoglycemia hyperthyroidism	acute/subacute
	tumor-related chorea		structural lesion	
	drug-induced		e.g. levodopa, lithium, amphetamine	
	infectious		e.g. HIV, toxoplasmosis	
	Sydenham chorea	childhood	autoimmune (group A beta-haemolytic Streptococcus infection)	
	Cerebral palsy	childhood	combination of genetic and neurometabolic causes	chronic

*- standard onset

#### Myoclonic dystonia

Rapid, lightning-like myoclonic jerks, which tend to be provoked by compound intentional movements, are the main manifestation of myoclonic dystonia with SGCE mutations. Such symptoms appear only in patients with this mutation. On the other hand, choreiform movements, characteristic of *NKX2-*1 mutation carriers, are absent in SGCE mutation carriers (Asmus et al. 2007).

### Treatment of neurological manifestations - summary of descriptions from previous reports

Tetrabenazine is recommended as a first-line pharmacological treatment for chorea (Patel and Jankovic 2014 [updated 2023]), starting with a low dose (0.5 mg/kg/day in children and 37.5 mg/day in adults divided into 2–3 doses). For pediatric patients, it was reported that increasing doses by 0.5 mg/kg at weekly intervals, depending on tolerability, up to a maximum of 4.5 mg/kg/day, without exceeding 100–150 mg/day, can be beneficial (Gras et al. 2012). Levodopa was reported in 9 cases as a successful treatment for involuntary movements (Farrenburg and Gupta 2020). The disruption of the *NKX2-1* gene causes abnormal migration of dopaminergic neurons in animal models (Kawano et al. 2003; Butt et al. 2008), which could explain the benefits of levodopa pharmacotherapy. Methylphenidate could significantly improve speech, motor skills, and gait in patients with coexisting ADHD diagnosis, even within 30 min after receiving a daily dose (Gauquelin et al. 2017). Another case described a young male with *NKX2-1* mutation and ADHD diagnosis who presented with generalized chorea. Methylphenidate considerably improved his symptoms (Tübing et al. 2018). In certain instances, multiple medications were used in combination or sequentially. The most common combinations included tetrabenazine with L-dopa, L-dopa with methylphenidate, and L-dopa with carbamazepine (Nou-Fontanet et al. 2023).

It was reported that l-thyroxine monotherapy improved stability and effectively managed drop attacks in a childhood-onset case of BHC. (Shiohama et al. 2018) Moreover, when l-thyroxine was suspended for a month, the drop attacks recurred despite the maintained euthyroidism in this patient. However, l-thyroxine did not improve the patient’s chorea (Shiohama et al. 2018). Another symptom responsive to treatment is dystonia. Proband (III-1) was effectively treated with botulinum toxin injections and DBS. It was also reported that myoclonus responded positively to levetiracetam in patients treated with small doses (Balicza et al. 2018) (Table 4).

**Table 4:** Summary of therapeutic options for the *NKX2-1*-related disorders

Manifestation	Methods of treatment	Description	References
Chorea	**Tetrabenazine**	0.5 mg/kg/day for children and 37.5 mg/day in adults	(Patel and Jankovic 2014 [updated 2023])
	**Levodopa**	2–6 mg/kg/day	(Farrenburg and Gupta 2020)
	**Methylphenidate**	20–72 mg/day	(Gauquelin et al. 2017), (Tübing et al. 2018)
Drop attacks	**L-thyroxine**	1–2 µg/kg/day	(Shiohama et al. 2018)
Dystonia	**DBS**	globus pallidus deep brain stimulation	This study
	**Botulinum toxin**	regular injections	(Balicza et al. 2018)
Myoclonus	**Levetiracetam**	2 × 12,5 mg	(Balicza et al. 2018)

Nou-Fontanet et al. (2023) summarized the usage of other medications (e.g. amantadine, beta-blockers, diazepam, olanzapine) that were described in isolated reports of patients with *NKX2-1-*related disorders. Additional research is necessary to assess the importance of these findings.

### Prognosis

Chorea tends to progress throughout puberty, then stabilizes in adulthood or even reducesin severity (Gras et al. 2012). Moreover, patients with *NKX2-1*-related disorders are expected to have a normal life expectancy (Fernandez et al. 2001). Our first proband’s follow-up revealed a stable disease course and unaffected life functioning.

## Discussion

*NKX2-1* gene mutations result in various and diverse phenotypes. Most analyzed cases presented the symptoms in infancy or early childhood, usually connected with delayed motor development, frequent falls, movement disorders, and compensated hypothyroidism. Therefore, *NKX2-1*-related disorders should be considered in the differential diagnosis of infants with such symptoms and family history. Lack of chorea does not always exclude the diagnosis of *NKX2-1*-related disorders, so the term “benign hereditary chorea” is not always accurate and should be avoided. Hereditary dystonias have one of the most complicated classifications. Some of them have distinct phenotypes, but overlap can always occur. In some cases, exact genetic diagnosis is important and may play a role in dystonia treatment (Weisheit et al. 2018).

The wide impact of the pathogenic *NKX2-1* variants on human development and neurological, endocrinological, and pulmonary diseases demonstrates the ongoing scientific challenges and issues. Despite discovering this gene locus and analyzing its regions, many details and processes regarding the pathogenesis and regulatory mechanisms of *NKX2-1*-related disorders remain unknown. A recent study showed that *NKX2-1* is expressed in certain types of lung adenocarcinoma resulting in unfavorable prognosis. Knockdown of *NKX2-1* causes suppression of cancer cell proliferation and, as a result, may be used as a molecular target in the treatment of lung adenocarcinoma (Matsubara et al. 2020). This is one example of the opportunities that targeted treatment might offer.

As described above, there is a wide spectrum of neurological cases with *NKX2-1* gene mutations, demonstrating a need for a thorough differential diagnosis in patients with chorea. Even HD and *NKX2-1*-related disorder are rare, and it seems unlikely that they occur in one family, but it is possible, as presented in this case. This case illustrates that a positive family history for HD does not exclude other causes of chorea as a possible diagnosis, including an *NKX2-1*-related disorder. Reporting novel mutations and cases of various phenotypes has an important potential value in fully understanding genetic diseases. The diversity among cases of *NKX2-1* patients and other neurogenetic disorders demonstrates the necessity of genetic testing to provide patients with molecularly targeted treatment in the future era of personalized medicine.

This study has several limitations. As described in the literature review section of the article, 81,2% of the analyzed patients had delayed motor milestones. Thus, they presumably presented with childhood-onset manifestations of the disease. However, it is a highly heterogeneous disorder that is a diagnostic challenge for clinicians, as well as a difficulty in determining whether all of the patients’ symptoms result from the *NKX2-1* gene mutation. Additionally, the report only includes data based on two families, and the true coincidence of the symptoms may be missed. Moreover, the pathogenicity of the newly reported variant was not confirmed in the functional analysis. The follow-up with some of the family members was missed. However, the report highlights the challenging diagnosis of genetic choreas in clinical practice.

## Data Availability

Data available on request due to restrictions e.g. privacy or ethical. The data presented in this study are available on request from the corresponding author. The data are not publicly available due to the patients’ privacy.
